# Effectiveness of Active Vision Therapy in Children with Bilateral Refractive Amblyopia

**DOI:** 10.18502/jovr.v21.18761

**Published:** 2026-07-21

**Authors:** Saeedeh Hoseinmenni, Saeed Rahmani, Ebrahim Jafarzadehpour, Jeffrey Cooper, Mohammad Reza Talebnejad, Ali Reza Akbarzade Baghban, Haleh Kangari, Enayatollah Osroosh

**Affiliations:** ^1^Department of Optometry, School of Rehabilitation Sciences, Shahid Beheshti University of Medical Sciences, Tehran, Iran; ^2^Department of Optometry, School of Rehabilitation Sciences, Iran University of Medical Sciences, Tehran, Iran; ^3^SUNY College of Optometry, New York, USA; ^4^Poostchi Ophthalmology Research Center, Department of Ophthalmology, School of Medicine, Shiraz University of Medical Sciences, Shiraz, Iran; ^5^Proteomics Research Center, Department of Biostatistics, School of Allied Medical Sciences, Shahid Beheshti University of Medical Sciences, Tehran, Iran

**Keywords:** Amblyopia, Bilateral Refractive Amblyopia, Vision Therapy

## Abstract

**Purpose:**

To compare the effectivity of vision therapy plus patching versus patching combined with placebo therapy in bilateral refractive amblyopia.

**Methods:**

In this randomized controlled trial, children with bilateral refractive amblyopia were enrolled. Monocular best-corrected visual acuity (BCVA) ranged from 0.3 logMAR (20/40) to 0.6 logMAR (20/80) in both eyes, and visual acuity (VA) difference was less than 0.2 logMAR between the eyes. The experimental group received patching plus in-office vision therapy with Vision Therapy System 4 (VTS4), including accommodation, stereopsis, ocular movements, and visual memory skills, while the control group received patching plus in-office placebo therapy. The primary outcome measure was BCVA, and the secondary outcomes were stereo acuity, and accommodative facility measured pre- and post-therapy.

**Results:**

Forty children aged 5 to 8 were included. After 4 months of therapy, the mean VA improvement was 0.23 ± 0.08 logMAR [2.3 line] (*P* < 0.001) in the experimental group and 0.09 ± 0.06 logMAR [0.09 line] (*P* < 0.001) in the control group. The difference in VA improvement between the two groups was statistically significant (*P* < 0.001). Post-therapy, the improvements in Randot stereoacuity and in monocular and binocular accommodative facilities were statistically significant between the experimental and the control groups (*P *= 0.013, *P *= 0.005, *P *= 0.002, respectively).

**Conclusion:**

Administering vision therapy in conjunction with patching significantly improved VA, stereo acuity, and accommodation facility in bilateral refractive amblyopia compared to patching alone.

##  INTRODUCTION

Amblyopia is a neurodevelopmental disorder that is caused by vision form deprivation during the critical period of visual development and affects the visual cortex. It is characterized by decreased visual acuity (VA) unilaterally or bilaterally.^[1]^ Amblyopia is often associated with conditions like strabismus, anisometropia, and/or refractive error. According to a recent study, the prevalence of amblyopia has been reported 1.36% (95% CI: 1.27 to 1.46%).^[2]^ The most common treatments include patching and atropine drops, but they do not address deficits in hand-eye coordination, stereo acuity, visual processing, and accommodation.^[3]^ Patching and atropine treatment are often marked by noncompliance, long treatment, and possible incomplete recovery of visual functions. A recent study found that while most patients with amblyopia had achieved full VA restoration, only 23.1% showed normal stereopsis.^[4]^ Therefore, it is important to consider alternative methods to restore other visual functions.^[5,6,7]^

Bilateral amblyopia is frequently associated with bilateral astigmatism or uncorrected high hyperopia before the age of two, and is marked by decreased best-corrected visual acuity (BCVA) in both eyes.^[4]^ The most common treatment is optical correction of the refractive error.^[8,9,10]^ Meanwhile, there is currently no standardized protocol for patching bilateral amblyopia,^[8,11]^ because it does not respond well to traditional treatment methods.^[12]^

An alternative method is vision therapy for isometropic amblyopia. In-office vision therapy includes anti-suppression techniques, sensory-neuro-motor exercises such as perceptual therapy, dichoptic therapy, video games, and virtual reality exercises which can be effective for improving VA, depth perception, and suppression in patients with amblyopia^[13,14,15]^ by activating neural channels in the visual cortex pathways.^[7,16]^ New vision therapy systems, such as Vision Therapy System Version 4 (VTS4), use liquid crystal glasses and offer computerized vision treatment. The liquid crystal glasses present stimuli alternately at 60 cps to each eye and, thus, are believed to minimize suppression, unlike anaglyphs, where color difference can increase suppression.^[17]^

Previous studies using dichoptic glasses and anti-suppression techniques have reported improved VA and depth perception in amblyopia.^[13,18,19,20]^ Children with amblyopia have a greater disposition to experiencing learning and reading difficulties.^[20]^ While patching alone may not be sufficient in eliminating binocular vision disorders, perceptual learning therapy has been reported to improve academic performance.^[21]^ Vision therapy has also been shown to reduce treatment duration.^[20,22]^ In traditional methods, recovery of VA is considered an endpoint of treatment for amblyopia; however, a recent study found that only 23.1% of children after full recovery of VA exhibited normal depth perception.^[4]^

Recent studies have employed dichoptic movies or digital games,^[18,19,20,23,24,25]^ but it seems these activities do not improve accommodation, vergence amplitudes, pursuit and saccadic eye movements, or hand-eye coordination. Despite the limited data and the predominance of retrospective research, vision therapy could offer benefits for bilateral amblyopia.^[11,20,23]^ Since most studies have focused on unilateral amblyopia,^[26,27,28]^ this study aimed to investigate the effectiveness of active vision therapy combined with patching versus patching alone in the treatment of bilateral refractive amblyopia.

##  METHODS

### Participants

Forty children aged 5–8 years were enrolled in this study. Each of the control and experimental groups comprised 20 individuals. All participants had bilateral amblyopia, defined as a decrease in monocular BCVA from 0.3 logMAR (logarithm of the minimum angle of resolution; 20/40) to 0.6 logMAR (20/80) in both eyes, and their VA difference was less than 0.2 logMAR between the eyes.^[8]^ All participants were hyperopic and/or astigmatic. The inclusion criteria were age 5–8 years, bilateral refractive amblyopia unresponsive to previous prescription, presence of residual amblyopia or use of new eyewear for at least 2 months without improvement in VA, BCVA of 0.3 logMAR (20/40) to 0.6 logMAR (20/80) in both eyes, and cycloplegic refractive error of > 3.00 D hyperopia and/or > 2.00 D astigmatism.^[4]^ None of the patients had previously undergone patching or vision therapy treatment.

Patients with any type of strabismus, or a phoria > 4 prism diopters (PD) at distance and near, were excluded. Children who were uncooperative during the tests or experienced nausea when using liquid crystal glasses were also excluded from the study.

The study was approved by the Ethics Committee of the Vice-Chancellor for Research and Technology of Shahid Beheshti University of Medical Sciences. Written informed consent was obtained from the parents of all patients.

During the first session, both uncorrected and best-corrected visual acuity (UCVA & BCVA) were measured using an LCD vision chart (HDC-9000N/PF, Huwitz Co., Anyang, South Korea) at near and at a distance of 4 meters. Measurements were recorded in logMAR units. Dry and cycloplegic refractions were conducted using an autorefractor (HRK-8000A, Huwitz Co., Anyang, South Korea). Cycloplegia was induced with two drops of cyclopentolate 0.5% at 5-minute intervals in each eye. Autorefraction measurements were taken 20 minutes after the second drop. In the second session, unilateral and alternate cover tests were performed.

Accommodative facility was measured with ± 2.00 D flipper lenses while the patients wore their distance correction. The lenses were flipped after the clarity of the vision, and the procedure was continued for 1 minute, and the number of cycles was recorded. The target was one line above the patient's near VA at 40 cm. The procedure was performed monocularly and binocularly and recorded as cycles per minute (cpm).

Stereoacuity was measured with the Randot Preschool Stereoacuity test (Stereo Optical Co., Inc., Chicago, IL, USA) and the Wirt Circles (Titmus Sterio test, Stereo Optical Co., Inc., Chicago, IL, USA). The Randot test was conducted using polarized glasses on the patient's eyes. Each page contains 8 depth perception targets. Initially, Page 1, which included depth perception levels of 100 and 200 seconds of arc, was presented to the patient. If the patient identified the targets, Page 2 (40 and 60 seconds of arc) was presented; otherwise, Page 3 (400 and 800 seconds of arc) was presented, and the results were recorded.

The Wirt Circles test consists of nine diamonds, with three diamonds in each row, and each diamond contains four circles with different retinal disparities. The patient wore polarized glasses over their correction (Stereo Optical Co., Inc., Chicago, IL, USA), and the level of depth perception was measured. The measured values ranged from 800 to 40 seconds of arc.

Prior to treatment, two groups were selected using the following criteria: age, spherical equivalent, and BCVA. Participants were then randomly assigned (four-piece block randomization, with 20 participants in each group) to either an experimental group that received 40 sessions of VTS4 training combined with patching or a control group that underwent patching with twice-monthly placebo therapy. The latter involved a simple digital movie using a liquid-crystal (LC) lens that was deactivated and had no influence on image perception, and a plano lens flipper to simulate accommodative exercises. In other words, participants engaged in noninterventional activities, including simple visual tasks and passive movie viewing, without the use of the VTS4. Although liquid crystal glasses were worn, the device was turned off and had no functional effect on the perceived images.

### Randomization Protocol

The participants were randomly assigned to either the experimental group or the control group through four-piece block randomization, with 20 participants in each group. Eight times, a random number between 1 and 6 was chosen to determine the selection of participants. The patients were not aware of their assigned group, and the main researcher was blinded to the group allocation of patients. The bock size was equal to 4 and finally 10 blocks were created.

### Blinding 

A double-masked approach was employed to minimize bias, meaning neither the main researcher nor the patients were aware of group assignments. A staff member assigned the participants into two groups (experimental and control) based on the block randomization list. A main researcher performed visual function tests pre- and post-therapy without knowing the group allocation of participants. Additionally, participants in the control group engaged in a simple digital movie as placebo therapy to further ensure blinding.

### Experimental Therapy Procedures

The patching regimen was 2 hours for each eye per day. The importance of adhering to the protocol was explained to the parents in both groups. To monitor compliance, parents' reports were checked by weekly follow-up phone calls to ensure that the protocol was being followed appropriately. According to these reports, both groups adhered to the patching protocol sufficiently. All patients adhered to the protocol of patching each eye for 1 to 2 hours per day. The vision therapy sessions were carefully administered by the therapist for the treatment group and were received well by the patients.

The experimental group underwent treatment using VTS4 (HTC Inc 2015, Version 1.7), which provides complex monocular and binocular stimuli. This system is able to measure and improve various visual skills such as pursuits, saccades, fusional amplitudes, motor fields, fixation disparity, accommodative facility, stereopsis, and visual memory. The computerized exercises in the VTS4 employed first-, second-, and third-degree targets, allowing patient interaction through use of a gamepad and mouse to adjust target demands based on their responses. The stimuli presentation used random dot stereograms (RDSs) in operant conditioning paradigm to improve either convergence or divergence fusional demands. RDSs require both bifoveal fixation and fusion to perceive stereopsis and lack monocular cues found in contour targets. Targets of varying sizes and disparities were presented, starting with larger targets and progressively moving to smaller ones. Liquid crystal glasses were used to separate the dichoptic stimuli to each eye. The glasses had a 15-foot effective radius, but normal viewing distance for VTS4 was 30 inches. The viewing distance was changed in case of administering the treatment at a distance other than 30 inches.

Sessions using the VTS4 lasted 30 minutes for at least 4 months. Initially, monocular accommodative rock exercises were performed in VTS4, followed by bi-ocular and then binocular tasks to increase accommodative amplitude and improve accommodative facility. Patients wore liquid crystal glasses during these exercises. Accommodative therapy progressed in difficulty using flipper lenses with increasing powers ( ± 0.5 D, ± 1.00 D, ± 1.50 D) to keep patients engaged and prevent fatigue. Each training session lasted 4-5 minutes. Flipper lenses were used to increase the demand on the accommodative system during the final stages of all following exercises.

Pursuit exercises consisted of nine levels that provided auditory feedback, with visual stimuli sizes adjusted according to the patient's VA —larger targets were used with patients who demonstrated lower acuity. For instance, patients tracked a dinosaur-like target moving in six different patterns while maintaining a rectangular frame around it. The liquid crystal glasses allowed one eye to view the dinosaur and the other to see the frame. This bi-ocular presentation was designed to reduce suppression and improve pursuit ocular movements. Each session lasted 4-5 minutes and included accommodative flippers in the later stages.

Saccade exercises used two types of targets: directional and E-letter targets, with sizes adjusted to match the patient's VA. Like the pursuit exercises, one eye viewed the target while the other observed the background, which could be modified using the keyboard. Similar to pursuits, anti-suppression therapy and accommodative flippers were used. Therapy lasted 4-5 minutes per session.

Visual memory was trained using directional arrows or Tumbling E targets, requiring patients to recall and indicate the direction of the targets that had been flashed. The flash duration varied from 10 to 0.1 seconds based on the child's performance. Targets were presented to each eye while participants wore liquid crystal glasses, facilitating engagement from both eyes in the memory task. These 4-5 minute exercises comprised part of the anti-suppression therapy, and accommodative flippers were added in the final stages.

Vergence exercises employed second-degree and stereoscopic vision targets to enhance fusional amplitudes at various distances and reduce suppression. In later phases, body movements were incorporated into the vergence exercises. The targets were presented at 6 meters and 1 meter. When the targets were viewed at 1 meter, the accommodative and vergence systems were integrated using flipper lenses. Anti-suppression exercises aimed at addressing peripheral and central suppression utilized shaking technology to improve functional skills.

After 4 months of training, we assessed VA, accommodative facility, and stereopsis.

### Statistical Methods for Data Analysis

Shapiro-Wilk test (SPSS version 26, IBM Corp., Armonk, NY, USA) was used to determine the normality of the data distribution. A marginal model utilizing Generalized Estimating Equations (GEE) was used to determine the intra-individual correlation between the right and left eyes. An unstructured method was used for correlation in the GEE analysis. Due to the non-normal distribution of binocular variable data, the Wilcoxon signed-ranks test was employed for within-group comparisons. In contrast, the Mann–Whitney U test was utilized to compare the two groups. A significance level of 0.05 was established, with *P*-values < 0.05 considered statistically significant.

##  RESULTS

**Figure 1:**
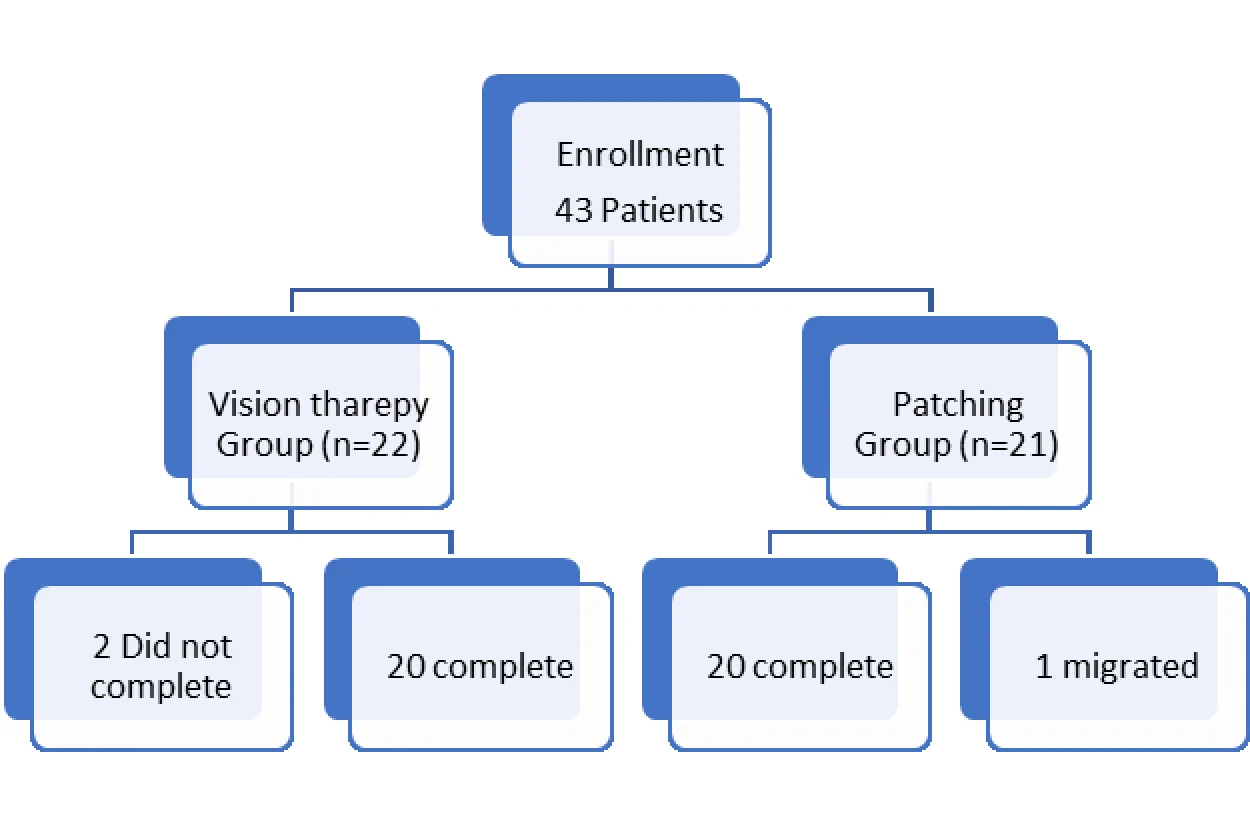
Flow diagram of the study participants.

Forty-three patients with bilateral refractive amblyopia were enrolled, and 40 patients completed therapy and were included in the statistical analysis. During the study, two patients discontinued vision therapy, and one patient in the control group became unavailable due to emigration [Figure 1]. The control and experimental groups each consisted of 20 individuals. The experimental group had a mean (SD) age of 6.10 ± 0.837 years (5 males, 15 females), while the control group had a mean (SD) age of 6.5 ± 1.147 years (11 males, 9 females).

The mean spherical equivalent and cycloplegic refractive error for the right eye (*P* = 0.723) and left eye (*P* = 0.495) were not significantly different between the two groups. The astigmatic error ranges were between 0.5 D and 5.00 D, and the hyperopic ranges between +2.00 D and +13.00 D.

Demographic information and baseline characteristics are presented in Table 1. Given the clustered nature of the data (as measurements of VA were taken from both eyes of each individual), a marginal model was applied using the GEE method for data analysis. All variables in both the experimental and control groups exhibited non-normal distribution.

**Table 1:** Demographic data and baseline characteristics of the participants

**Baseline characteristics of the participants**		**Experimental group**	**Control group**	***P*** **-value**
Age		6.10 ± 0.837	6.50 ± 1.147	0.216
Gender		5M/15F	11M/9F	0.053 ${}^{*}$
Equivalent sphere	OD	4.76 ± 2.72 (1.25 to 12.75)	4.43 ± 3.13 (0.00 to 12.00)	0.723
	OS	5.09 ± 2.89 (1.75 to 13.00)	4.47 ± 2.78 (0.25 to 9.00)	0.495
${}^{*}$ *P*-value based on the chi-square test.				

Table 2 illustrates the changes in mean, maximum, and minimum BCVA before treatment and 4 months after in both groups. There was no statistically significant difference in VA between the two groups at baseline, and the two groups were homogeneous before the intervention (*P *= 0.501). The Wilcoxon test was used for group comparisons. After the intervention, however, the experimental group (vision therapy and patching) demonstrated a statistical difference (*P* < 0.001) which was also clinically significant. Inter-group analysis using the Mann–Whitney test found statistically significant mean differences (*P* < 0.001), demonstrating that vision therapy was more effective than patching.

**Table 2:** Intra- and inter-group comparisons of visual acuity in the groups

			**Experimental group**	**Control group**	***P*** **-value**
Visual acuity LOG Mean ± SD (min to max)	Before treatment	OD	0.37 ± 0.08 (0.30 to 0.50)	0.35 ± 0.06 (0.30 to 0.54)	0.501
		OS	0.39 ± 0.10 (0.3 to 0.60)	0.37 ± 0.08 (0.30 to 0.50)	
	After treatment	OD	0.14 ± 0.08 (0.00 to 0.30)	0.26 ± 0.09 (0.10 to 0.50)	0.001
		OS	0.14 ± 0.08 (0.00 to 0.40)	0.27 ± 0.10 (0.10 to 0.50)	
		*P*-value	< 0.001	< 0.001	

Table 3 illustrates changes in the mean, maximum, and minimum of stereoacuity before treatment and at 4 months after treatment in both groups. The Mann–Whitney test comparing the two groups' stereoacuity measured with Wirt Circles and Randot showed no significant difference at baseline (*P* = 0.359, *P* = 0.968, respectively).

Within-group comparisons of Wirt Circle and Randot stereoacuity in both the experimental and control groups confirmed the effect of treatment (*P* = 0.001, *P* = 0.002, *P* = 0.04, *P* = 0.025, respectively). Inter-group analysis using the Mann–Whitney test showed statistically significant mean differences in Randot stereoacuity (*P* = 0.013). In contrast, the difference in mean Wirt Circle stereoacuity between the two groups was not statistically significant, although the *P*-value approached significance (*P* = 0.051).

**Table 3:** Intra- and inter-group comparisons of stereoacuity

			**Experimental group**	**Control group**	***P*** **-value**
Stereo acuity LOG Mean ± SD (min to max)	Wirt Circle	Before treatment	2.18 ± 0.37 (1.78 to 2.90)	2.18 ± 0.37 (1.70 to 2.90)	0.968
		After treatment	1.90 ± 0.18 (1.60 to 2.30)	2.12 ± 0.40 (1.70 to 2.90)	0.051
	*P*-value		0.001	0.04	
	Randot	Before treatment	2.44 ± 0.47 (1.78 to 2.90)	2.60 ± 0.36 (2.00 to 2.90)	0.369
		After treatment	2.21 ± 0.40 (1.78 to 2.90)	2.52 ± 0.36 (2.00 to 2.90)	0.013
*P*-value			0.002	0.025	

Table 4 presents changes in the mean, maximum, and minimum of the accommodative facility before treatment and 4 months after in both groups. Comparing monocular and binocular accommodative facility in the two groups before treatment, the Mann–Whitney test yielded no significant differences (*P* = 0.139 and *P* = 0.359, respectively). Within-group comparisons supported that both monocular and binocular accommodative facility improved in both the experimental and control groups relative to baseline (*P* = 0.001, *P* = 0.001, *P* = 0.002, *P* = 0.001, respectively). According to Table 4, intergroup analysis indicated that treatment effects were statistically significant for both monocular and binocular accommodative facilities (*P *= 0.005, *P* = 0.002, respectively).

**Table 4:** Intra- and inter-group comparisons of accommodative facility

			**Experimental group**	**Control group**	***P*** **-value**
Accommodative facility (cycle per minute)	Before treatment	OD	4.40 ± 0.14 (1.50 to 7.50)	3.72 ± 0.13 (1.00 to 5.50)	0.139
		OS	4.27 ± 0.18 (1.50 to 8.50)	3.82 ± 1.29 (1.50 to 6.00)	
	After treatment	OD	5.92 ± 1.48 (2.00 to 8.00)	4.37 ± 1.31 (2.00 to 7.00)	0.005
		OS	6.05 ± 1.42 (3.5 to 9.00)	4.57 ± 1.44 (2.00 to 6.50)	
		*P*-value	0.001	0.002	
	Before treatment	OU	3.72 ± 0.99 (1.50 to 6.00)	3.52 ± 1.24 (2.00 to 6.50)	0.359
	After treatment	OU	4.40 ± 0.14 (1.50 to 7.50)	4.20 ± 1.35 (2.00 to 8.00)	0.002
		*P*-value	0.001	0.001	

**Figure 2:**
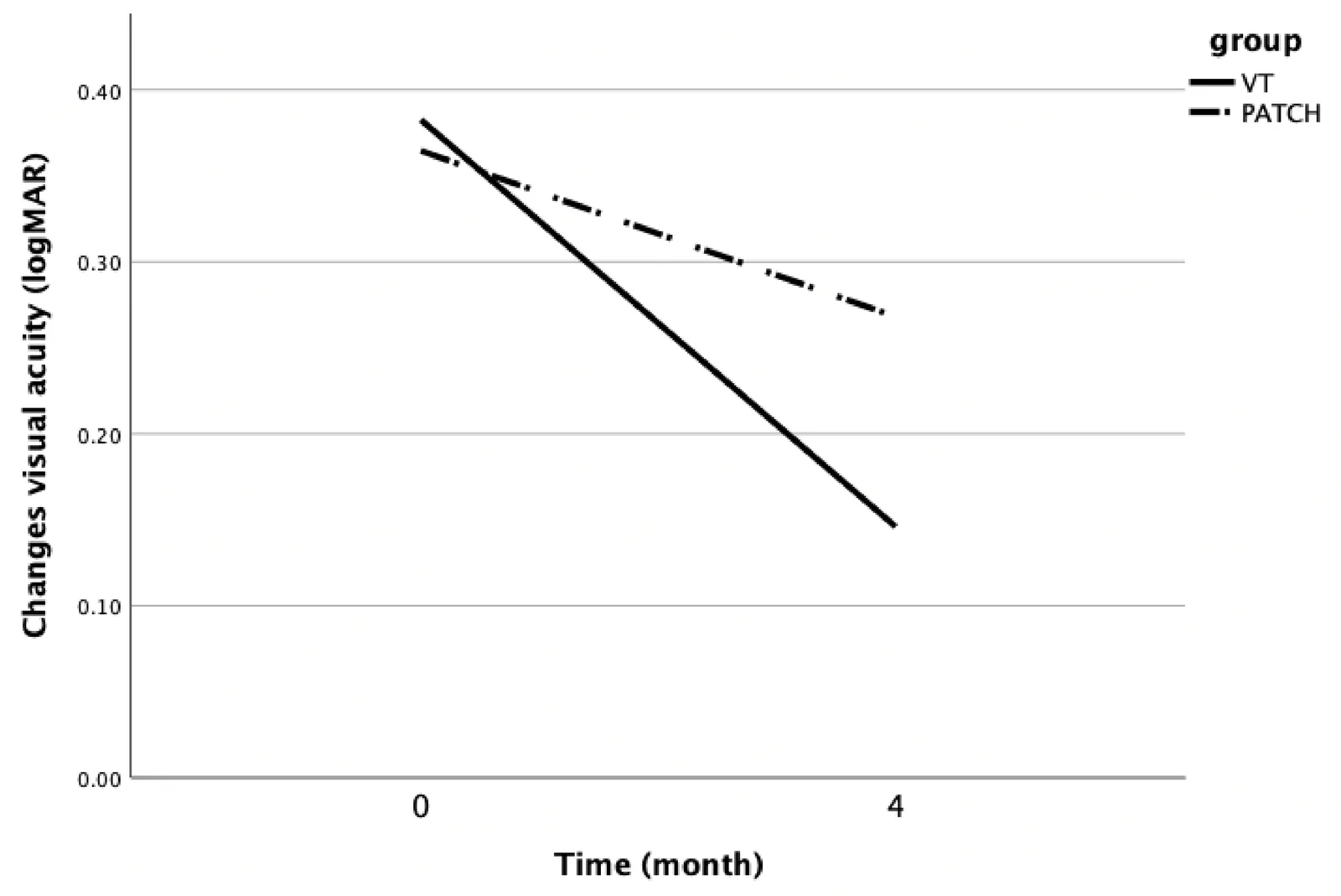
Changes in the mean visual acuity before and after treatment in the vision therapy plus patching group versus patching with placebo therapy group.

Figure 2 illustrates the mean changes in logMAR VA before treatment and 4 months later in both groups (vision therapy plus patching group and patching with placebo therapy). Further analysis of the model coefficients revealed that for each one-unit increase in the post-treatment period, the slope of logMAR VA declined by –0.02 units in the control group and –0.06 units in the experimental group.

Tables 5 and 6 present the results of the GEE analysis model fitting for VA and monocular accommodative facility, respectively. The columns are interrelated to ultimately determine the statistical significance of the variables' effects. In sum, the data in the tables show that the rate of improvement in the experimental group is significantly better than in the control group.

**Table 5:** The results of fitting the GEE model to visual acuity

**Source**	**B**	**Standard error**	**Wald Chi-square **	***P*** **-value**
Intercept	0.361	0.022	274.038	0.001 >
${}^{*}$ Group	0.02	0.03	0.453	0.501
Time	–0.02	0.006	12.31	0.001 >
Group ${}^{*}$ time	–0.04	0.008	17.49	0.001 >
${}^{*}$ The control group is the reference group. B, estimated coefficient of variables; SE, standard error of the coefficient; Wald Chi-square, test statistic for significance; Intercept, mean of dependent variable in control group at baseline; Group, difference between groups at baseline; Time, slope of change over time in control group; Time + Group*Time, slope of change over time in intervention group; *P*-value, significance level of the coefficient.				

**Table 6:** The results of fitting the GEE model to monocular accommodative facility

**Source**	**B**	**Standard error**	**Wald Chi-square **	***P*** **-value**
Intercept	3.79	0.29	173.79	0.001 >
${}^{*}$ Group	0.606	0.409	2.188	0.139
Time	0.178	0.047	14.04	0.001 >
Group ${}^{*}$ time	0.261	0.092	8.00	0.005
${}^{*}$ The control group is the reference group. B, estimated coefficient of variables; SE, standard error of the coefficient; Wald Chi-square, test statistic for significance; Intercept, mean of dependent variable in control group at baseline; Group, difference between groups at baseline; Time, slope of change over time in control group; Time + Group*Time, slope of change over time in intervention group; *P*-value, significance level of the coefficient.				

**Figure 3:**
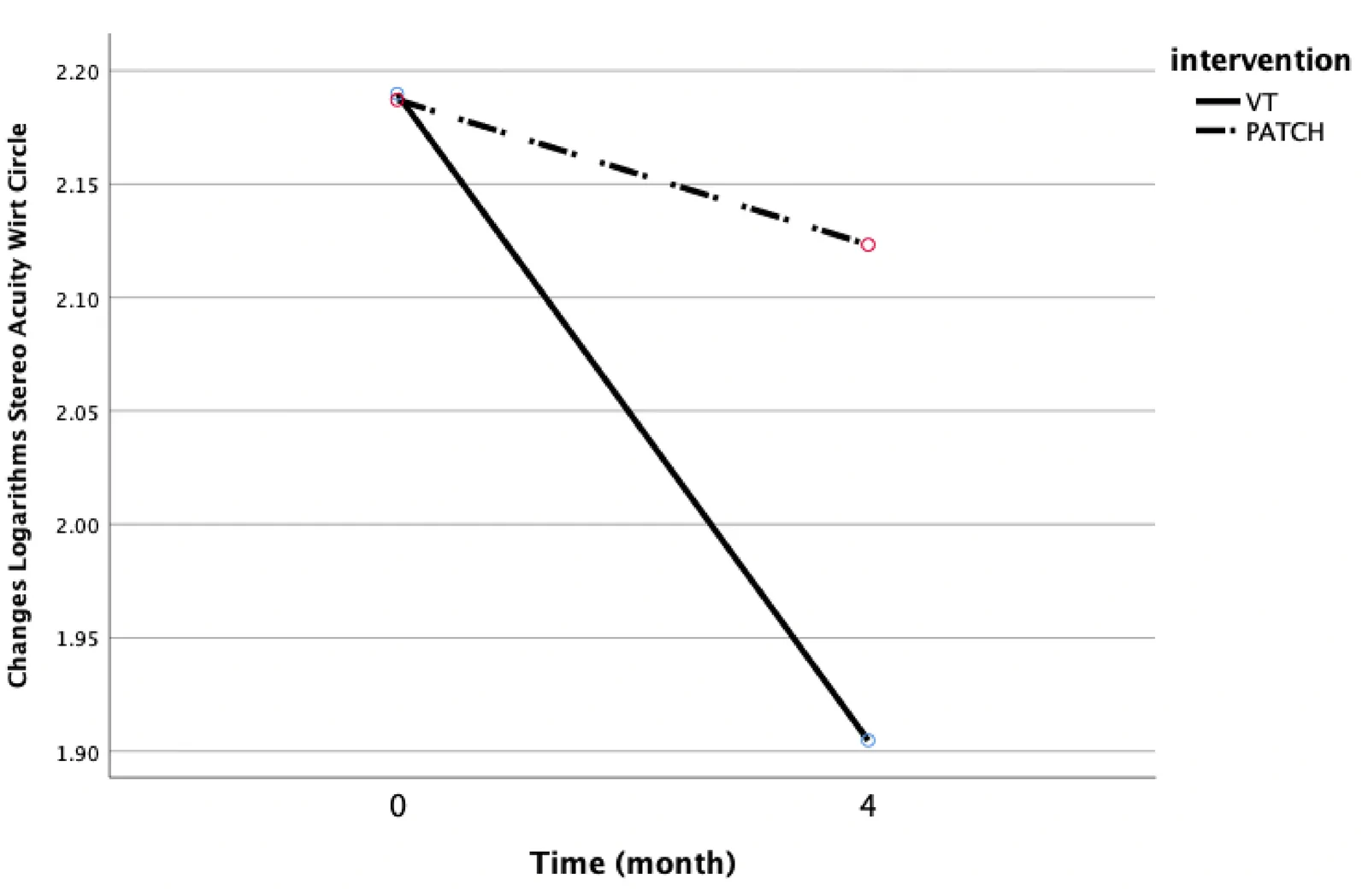
Changes in the mean Wirt Circle stereoacuity (Log) before and after treatment in the vision therapy plus patching group versus the patching with placebo therapy group.

According to Figure 3, the reported mean logarithmic stereoacuity for the Wirt Circle test in the experimental group was 2.1897 before treatment and 1.9047 post-treatment, indicating an improvement of approximately 0.28 logarithm. For the control group, this value was 2.1871 before treatment and 2.1233 after treatment, showing an improvement of 0.06 logarithm. Figure 3 also illustrates the changes in the mean logarithm of stereoacuity in the Wirt Circle test before treatment and 4 months after in both groups. The post-treatment results of the two groups were compared using the Mann–Whitney test, revealing a significant statistical difference between the two groups after treatment (*P* = 0.051).

**Figure 4:**
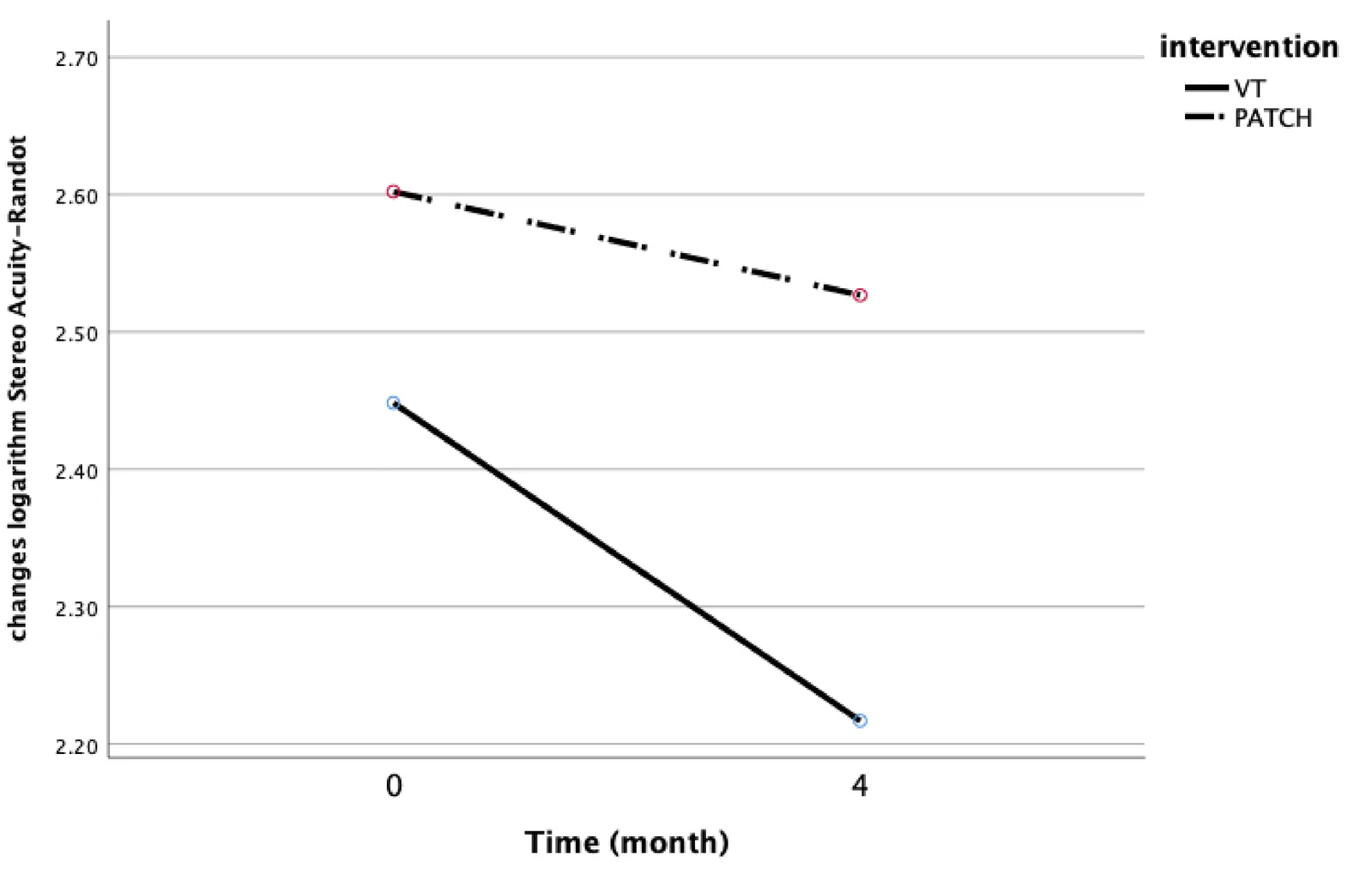
Changes in the mean Randot Stereoacuity (Log) before and after treatment in the vision therapy plus patching group versus the patching with placebo therapy group.

Figure 4 illustrates the mean logarithmic stereoacuity values obtained using the Randot test for the experimental group, which were 2.4484 before treatment and 2.2169 after treatment, indicating an improvement of approximately 0.23 arcsec (*P *= 0.002). In the control group, this value was 2.6021 before treatment and 2.5268 after treatment, showing an improvement of 0.08 (*P* = 0.025). Figure 4 displays changes in mean logarithmic stereoacuity on the Randot test before treatment and 4 months after treatment in both groups. The results using the Mann–Whitney test indicate a statistically significant difference between the two groups after treatment (*P* = 0.013).

**Figure 5:**
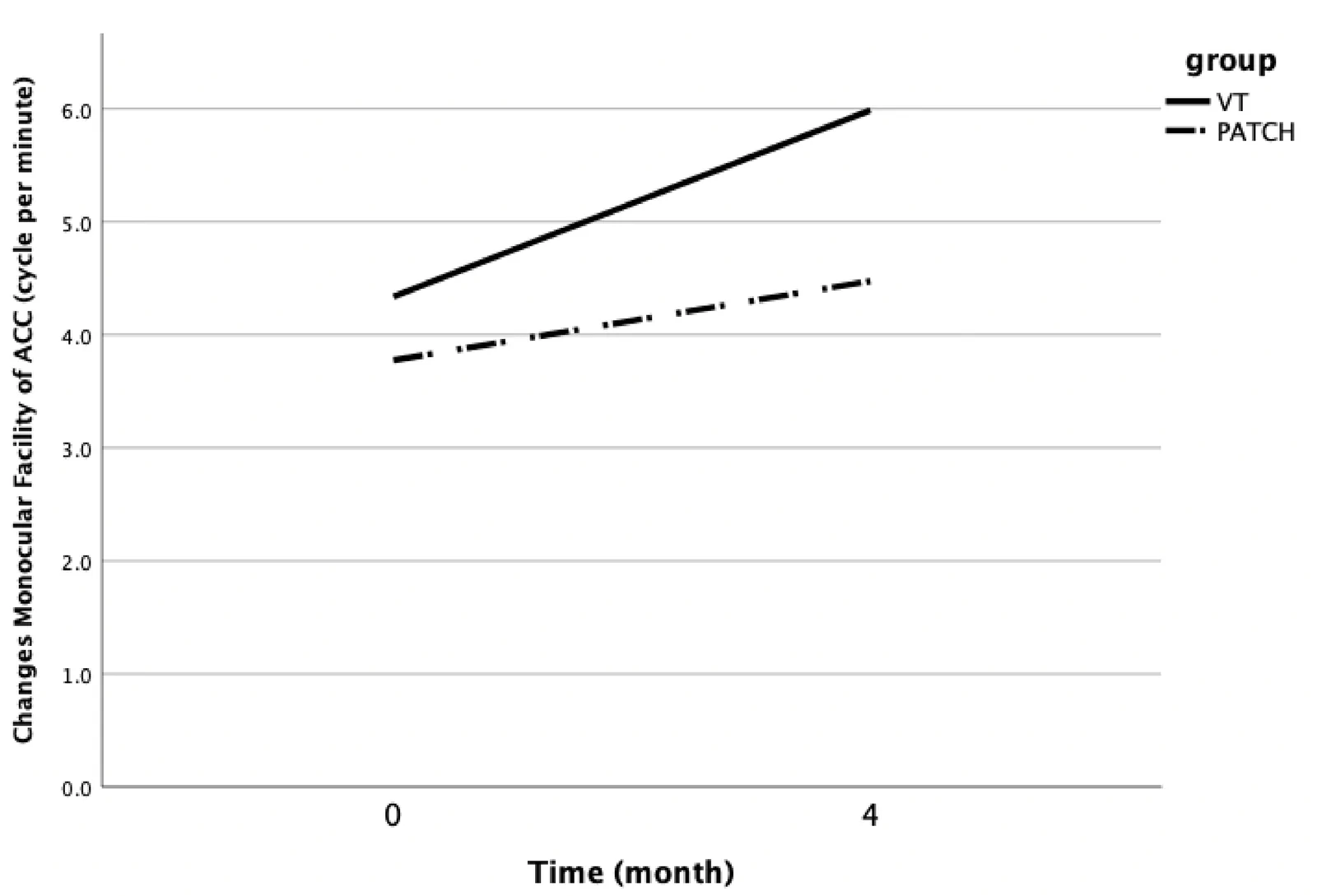
Changes in the mean monocular accommodative facility (cpm) before and after treatment in the vision therapy plus patching group versus patching with placebo therapy group.

Figure 5 illustrates the mean monocular accommodative facility changes for both the experimental and control groups at two time points: before treatment and 4 months after. The estimated coefficients from the GEE model indicated that, in the control group, the mean monocular accommodative facility increased by 0.18 cycles/min for each month of training. In contrast, the experimental group showed an increase of 0.44 cycles/min for each month of training.

**Figure 6:**
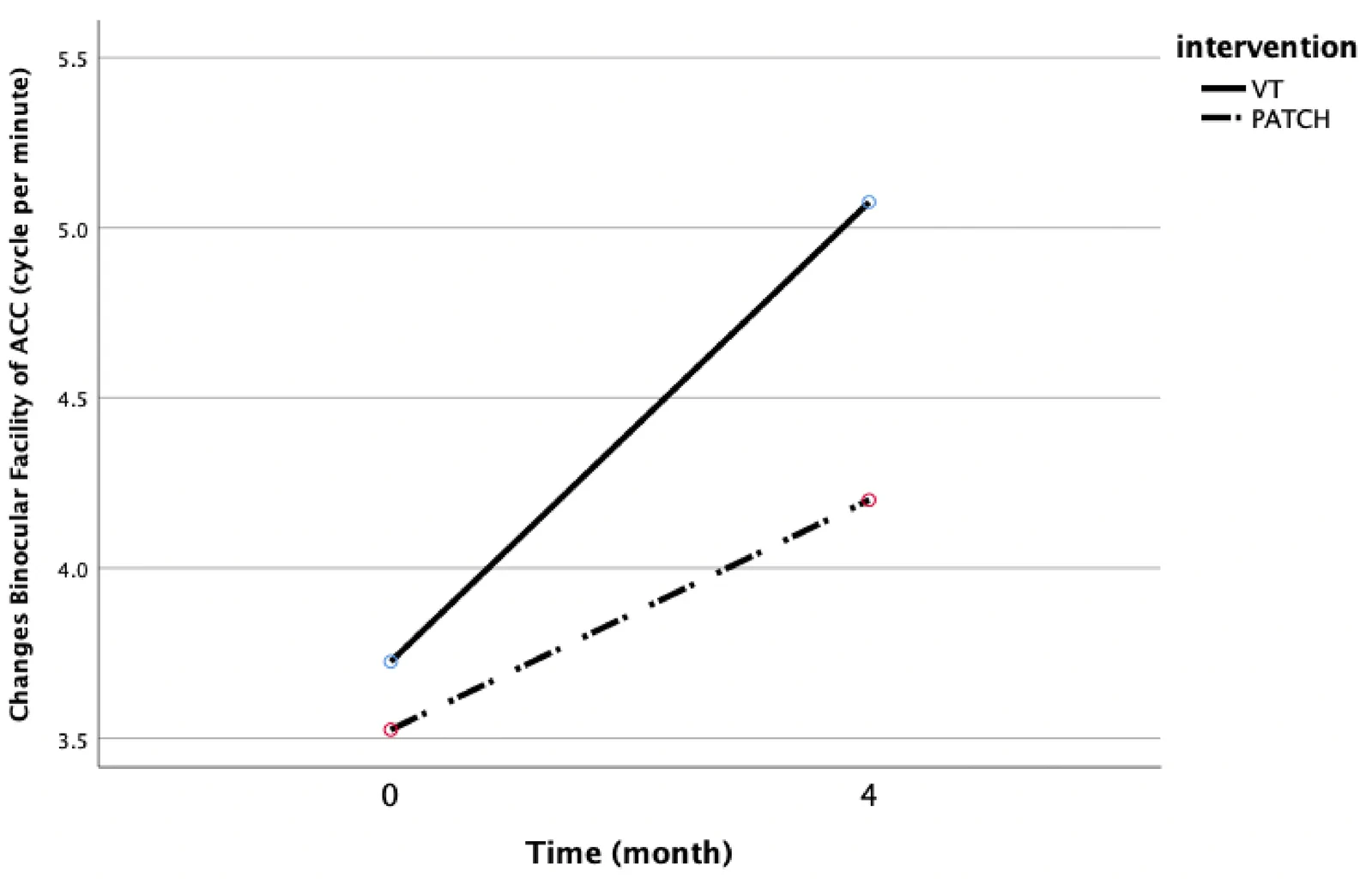
Changes in the mean binocular accommodative facility (cpm) before and after treatment in the vision therapy plus patching group versus the patching with placebo therapy group.

Figure 6 illustrates the mean changes in binocular accommodative facility for both groups before and 4 months after treatment. The experimental group demonstrated a mean increase in binocular accommodative facility, as supported by the steeper slope. Both groups showed a significant improvement of 1.3 cycles/min, and the control group showed a significant increase of 0.7 cycles/min in the mean binocular accommodative facility. An inter-group comparison after treatment showed a significant difference between groups (*P* = 0.002). Therefore, the two methods are effective, although vision therapy plus patching was more effective.

##  DISCUSSION

Our findings demonstrated a significant intra-group difference in VA, stereoacuity, and accommodative facility 4 months post-treatment between the vision therapy plus patching group and the patching with placebo therapy group. The inter-group differences were also significant, and the rate of improvement in the experimental group was nearly three times that of the control group.

Our results indicated the significant impact of visual training on improving VA in the experimental group. As far as the experimental group is concerned, our findings are in agreement with those of a retrospective study that included 161 young patients (aged 4–13 years) with anisometropia and isoametropia. That study reported improvements in VA up to 0.39 logMAR after 6 weeks of training using the Bynocs software and red–green anaglyph glasses.^[22]^ In another retrospective study, Huang et al treated patients aged 7–10 years with anisometropic and isoametropic amblyopia, and they reported an improvement in VA with visual training (mean logMAR: 0.32 ± 0.15) versus 0.003 ± 0.19 with patching after 6 months. In that study, patching was used for 4 hours per day only for the amblyopes who demonstrated an interocular difference of 2 lines or more.^[20]^ These studies reported greater improvement in BCVA following active treatment than our study (2.4 lines), which could be due to different patient characteristics. These studies suggest that Ogle's patent stereopsis with diplopia vision therapy results in faster and better treatment outcomes.

Some researchers believe that binocular treatment should not be advocated to replace patching.^[29]^ Others have shown that there is no difference between using optical correction versus patching, concluding that only optical correction is necessary in the treatment of amblyopia.^[8]^ Some have suggested that occlusion is only needed when anisometropia exists in bilateral amblyopia and the dominant eye improves to VA of 20/30.^[10]^ Overall, no standard guidelines have been established for patching.^[8]^

Some studies indicated that spectacle correction alone may improve bilateral amblyopia over a long period. For example, in a multicenter retrospective study in 2004 by the Pediatric Eye Disease Investigator Group, Klimec et al reported that 83% of patients with bilateral refractive amblyopia who only used glasses reached a VA threshold of 0.3 logMAR (20/40) after 3 years, and only 48% improved to 0.1 logMAR (20/25).^[9]^ Also, in a 2007 retrospective study involving 161 amblyopic children (31 had isoametropia) treated with optical correction alone, Ziylan et al reported that 83.9% of patients had BCVA > 0.5 (Snellen units) at the last visit. Almost half the patients (48.8%) had BCVA ≥ 0.8 at the last visit over approximately 5 years.^[30]^ Another study in 2007 by Wallace et al, which included 113 children with an average age of 5.1 years, noted that binocular VA of 0.1 logMAR or better was achieved after approximately 2 years of wearing glasses.^[11]^ It seems achieving the 0.1 logMAR threshold through optical correction alone requires a long treatment period in the case of bilateral refractive amblyopia. These studies emphasize spectacle correction alone, whereas the control group in our study—patients with bilateral refractive amblyopia—experienced more significant changes in VA after 4 months of optical correction and patching. The results of the current study showed a significant improvement in VA in the experimental group, which helps promote the quality of life of patients. Meanwhile, vision improvement in the control group was statistically significant, but not clinically noticeable.

In the Wirt Circle stereoacuity test (line stereogram), both the experimental and control groups showed relative intra-group improvement in stereoacuity. Comparing the two groups after treatment revealed a trend, and if our sample size were larger, this difference could have become significant. In the Randot test, an RDS, the findings indicated that intra- and inter-group improvements in stereoacuity were statistically significant. A larger positive effect was noted with line stereogram than RDS in the experimental group compared to the control group.

There are slightly different mechanisms behind line stereograms and RDS tests. The Randot test is composed of global cues without lines and edges, while the Wirt test uses contour and edges (lines), which are believed to require different processing.^[31]^ In a study of healthy individuals, the stereoacuities were similar between RDS and line stereograms.^[32]^ Brich et al showed that the findings for these two tests were the same when the stimuli were better than 160 arc seconds.^[33]^ Cooper and Warchowsky showed that monocular cues are only available in 60 arcsec or worse and not below 40 arcsec.^[34]^ It is still unclear whether the overestimation of the Wirt Circles stereoacuity test is due to its neural mechanism or monocular cues.^[32]^

Aligned with the present study, several studies have also reported significant improvements in binocular function in children aged 4 to 13 years following dichoptic training.^[22]^ In one study involving patients aged 7 to 10 with bilateral amblyopia no longer responsive to occlusion, depth perception improved by approximately 55.26% after 6 months of dichoptic training.^[20]^ Stereoacuity provides a precise measurement for assessing binocular function.^[35]^ All active training techniques aim to amplify the signal and eliminate suppression, ultimately aiming to reduce amblyopia.

In our study, stereoscopic training combined with in-office vision therapy enhanced the patients' depth perception more than patching alone. Using anti-suppression exercises and exposing patients to stimuli with varying disparities can be effective, even when initial stereoscopic depth perception is poor. We believe that vision therapy leads to greater stimulation of neural pathways and improved information processing in the visual cortex.^[20,36,37]^ Furthermore, in amblyopia, visual deprivation may cause inactivation of layers within the lateral geniculate nucleus, thereby reducing visual function, including reduced contrast sensitivity, VA, and stereoacuity. Conversely, exposure to appropriate visual stimuli can facilitate enhancement and the formation of new neural connections.^[38,39]^ Our findings illustrated the importance of active treatment, such as repeated exposure to stimuli with varying disparities during visual therapy, to improve depth perception. While occlusion therapy also yields improvements in depth perception, its effects may be less significant than visual training techniques. For example, Liu reported a 27% enhancement in stereoacuity, which is higher than the 50% improvement noted in earlier studies that had employed monocular techniques.^[40]^

Our results also indicated that both monocular and binocular accommodative facilities increased considerably in the two groups post-treatment. Nevertheless, 20% of participants in the experimental group and 60% in the control group showed no significant changes in accommodative facility before and after treatment. Additionally, the slope of improvement over time was significantly steeper in the experimental group than in the control group. It should be noted that the improvement in the monocular accommodative facility (1.6–1.8 cpm) in the experimental group brought patients closer to age-appropriate norms, which will likely help to enhance near-vision functions such as reading and other close activities. Previous research involving similar patient populations has not explored the influence of accommodative training.^[20,22]^ Our findings suggest that using accommodative exercises with various lens powers in a dichoptic setting, combined with different target sizes, may activate neural pathways related to accommodation in cases of amblyopia.

While studies on bilateral amblyopia remain scarce, our study provides further support that active vision therapy improves the outcome of amblyopia treatment.^[20,22]^ Similar to the research by Papageorgiou, our findings show that vision therapy helps improve VA, eliminate suppression, and foster neuroplasticity within the visual system.^[41]^

The limitations of this study include a small sample size and poor compliance with patching in both groups.

In summary, this research demonstrated that in moderate bilateral amblyopia, patching plus active vision therapy was more effective than patching alone in terms of improving VA, stereoacuity, and accommodative facility.

##  Financial Support and Sponsorship

None.

##  Conflict of Interest

None.
